# Molecular Dissection of Seedling Salinity Tolerance in Rice (*Oryza sativa L*.) Using a High-Density GBS-Based SNP Linkage Map

**DOI:** 10.1186/s12284-016-0125-2

**Published:** 2016-10-01

**Authors:** Teresa B. De Leon, Steven Linscombe, Prasanta K. Subudhi

**Affiliations:** 1 School of Plant, Environmental, and Soil Sciences, Louisiana State University Agricultural Center, Baton Rouge, LA USA; 2 Rice Research Station, Louisiana State University Agricultural Center, Rayne, LA USA

**Keywords:** *Oryza sativa*, Genotyping by sequencing, Quantitative trait locus, Salt tolerance, Candidate gene, Single nucleotide polymorphism

## Abstract

**Background:**

Salinity is one of the many abiotic stresses limiting rice production worldwide. Several studies were conducted to identify quantitative trait loci (QTLs) for traits associated to salinity tolerance. However, due to large confidence interval for the position of QTLs, utility of reported QTLs and the associated markers has been limited in rice breeding programs. The main objective of this study is to construct a high-density rice genetic map for identification QTLs and candidate genes for salinity tolerance at seedling stage.

**Results:**

We evaluated a population of 187 recombinant inbred lines (RILs) developed from a cross between Bengal and Pokkali for nine traits related to salinity tolerance. A total of 9303 SNP markers generated by genotyping-by-sequencing (GBS) were mapped to 2817 recombination points. The genetic map had a total map length of 1650 cM with an average resolution of 0.59 cM between markers. For nine traits, a total of 85 additive QTLs were identified, of which, 16 were large-effect QTLs and the rest were small-effect QTLs. The average interval size of QTL was about 132 kilo base pairs (Kb). Eleven of the 85 additive QTLs validated 14 reported QTLs for shoot potassium concentration, sodium-potassium ratio, salt injury score, plant height, and shoot dry weight. Epistatic QTL mapping identified several pairs of QTLs that significantly contributed to the variation of traits. The QTL for high shoot K^+^ concentration was mapped near the *qSKC1* region. However, candidate genes within the QTL interval were a CC-NBS-LRR protein, three uncharacterized genes, and transposable elements. Additionally, many QTLs flanked small chromosomal intervals containing few candidate genes. Annotation of the genes located within QTL intervals indicated that ion transporters, osmotic regulators, transcription factors, and protein kinases may play essential role in various salt tolerance mechanisms.

**Conclusion:**

The saturation of SNP markers in our linkage map increased the resolution of QTL mapping. Our study offers new insights on salinity tolerance and presents useful candidate genes that will help in marker-assisted gene pyramiding to develop salt tolerant rice varieties.

**Electronic supplementary material:**

The online version of this article (doi:10.1186/s12284-016-0125-2) contains supplementary material, which is available to authorized users.

## Background

Rice is a staple food crop for many countries in Asia, Africa, and Latin America. In spite of increased production worldwide, rice growers are faced with challenges caused by both biotic and abiotic stresses. Hence, breeding programs targeted to address those problems are implemented. Among the abiotic stresses, soil and water salinity is a problem not only in the coastal areas but also in areas where crop production heavily relies on irrigation with poor drainage system. Previous studies have indicated that rice is sensitive to salt stress during seedling stage and reproductive stage (Pearson and Bernstein 1959; Zeng et al. 2001). Rice seedlings wither and eventually die at 10dSm^-1^ salt stress (Munns et al. 2006) while yield loss can be as high as 90 % at 3dSm^-1^ salt level (Asch et al. 2000). Progress in breeding rice with salt tolerance is slow due to genetic complexity of salinity tolerance (Flowers and Flowers 2005). Some germplasms with high salt tolerance are available. However, majority of these germplasms possess many undesirable traits. Pokkali, Nona Bokra, and Hasawi, which are highly tolerant and often used as donors in breeding for salt tolerance, are tall, photosensitive, low yielding, and have red kernel. In addition, salt tolerance screening is difficult because the phenotypic response of rice to salt stress is highly affected by other confounding environmental factors (Gregorio and Senadhira 1993; Flowers 2004). Hence, the search for QTLs and DNA markers tightly linked to traits related to salt tolerance becomes a major objective in most breeding programs. It is assumed that molecular markers will facilitate a fast and cost-effective screening of large populations (Munns and James 2003).

Since the advent of molecular markers, QTL analyses for salinity tolerance at seedling stage were conducted using RIL (Koyama et al. 2001; Gregorio et al. 2002; Wang et al. 2012), F_2:3_ lines (Lin et al. 2004), and backcross populations (Thomson et al. 2010; Alam et al. 2011). QTLs for visual scoring, survival, shoot and root lengths, Na^+^/K^+^ ratio, Na^+^ and K^+^ concentrations, in root and shoot were frequently investigated at 100–120 mM salt stress. Most of the QTL mapping studies have indicated polygenic nature of salinity tolerance. Among the QTLs for traits related to salt tolerance, only *qSKC1* was successfully isolated by map-based cloning (Ren et al. 2005). The *SKC1* gene from Nona Bokra encodes an HKT-type transporter that regulates the Na^+^/K^+^ homeostasis under salt stress. In earlier reports, the QTL designated as *Saltol* (Gregorio 1997) and a gene ‘*SalT’* (Causse et al. 1994) for Na^+^/K^+^ ratio were located on chromosome 1.

Numerous QTL mapping studies for salinity tolerance were based on linkage maps constructed using AFLP (Gregorio 1997), RFLP (Koyama et al. 2001; Bonilla et al. 2002; Lin et al. 2004), and SSR markers (Thomson et al. 2010; Wang et al. 2012). The population size was usually small and the markers were sparse due to limited polymorphism between the parents. The rapid development in the sequencing technology makes single nucleotide polymorphism (SNP) to become the marker of choice for QTL mapping. Bimpong et al. (2013) used 194 polymorphic SNP markers for mapping QTLs related to salinity tolerance. More recently, Kumar et al. (2015) applied the genome-wide association (GWAS) mapping on 220 rice varieties using a custom-designed array containing 6000 SNPs. Major association of Na^+^/K^+^ ratio still co-localized to the *Saltol* locus with additional QTLs on chromosome 4, 6, and 7. Significant SNPs were identified and some candidate genes were suggested. However, tight association of candidate genes in or around a single variant still needs enrichment with more markers at a locus to avoid false association. Moreover, complete resequencing of the locus in tolerant and non-tolerant lines or in bi-parental population are needed to add credence to the robustness of GWAS using SNP array.

The introduction of genotyping-by-sequencing (GBS) and the availability of whole genome sequence of rice have accelerated the identification of millions of SNPs across the whole genome. To date, GBS is becoming popular for population studies, genetic diversity, QTL mapping, and genomic selection (He et al. 2014). GBS enabled the construction of high-density linkage map and QTL analysis in maize, wheat, barley (Poland et al. 2012; Chen et al. 2014), oat (Huang et al. 2014), and chickpea (Jaganathan et al. 2015). In rice, GBS has been applied in QTL mapping for leaf width and aluminum tolerance (Spindel et al. 2013), pericarp color and some agronomic traits (Arbelaez et al. 2015), and rice blast resistance (Liu et al. 2015). Several QTL mapping studies for salinity tolerance have been reported. However, QTLs and markers flanking QTLs for salinity tolerance are not being utilized in breeding programs. The main reason for this is attributed to the large chromosome intervals delimited by those QTLs. Thus, identification of candidate genes and understanding of salinity tolerance mechanism still remained a challenge.

In this study, a recombinant inbred line population at F_6_ generation, developed from the cross Bengal x Pokkali, was used. Bengal is a high yielding, early maturing, semi-dwarf, medium grain cultivar developed from the cross of MARS//M201/MARS (Linscombe et al. 1993). It is sensitive to salinity stress (De Leon et al. 2015). Pokkali is a highly tolerant landrace often used as a donor for salinity tolerance. However, it is notable for many undesirable traits such as low-yield, tall, and highly susceptible to lodging. It is photoperiod-sensitive, awned, with red pericarp and poor cooking quality (Gregorio et al. 2002). We used the GBS technique to construct a high-resolution genome-wide SNP genetic map for identification of additive and epistatic QTLs for salinity tolerance. Segregation distortion loci (SDLs) and QTLs for plant height were mapped to show the quality and accuracy of the genetic map and QTL mapping. Our ultra-high density map allowed us to map QTLs with high resolution and identify candidate genes that may play important role in the mechanism of salt tolerance in rice. The candidate genes identified in this study will serve as useful targets for functional genomics, gene pyramiding, and for gene-based marker-assisted breeding for salinity tolerance.

## Results

### Phenotypic Characterization Under Salt Stress

**Fig. 1:**
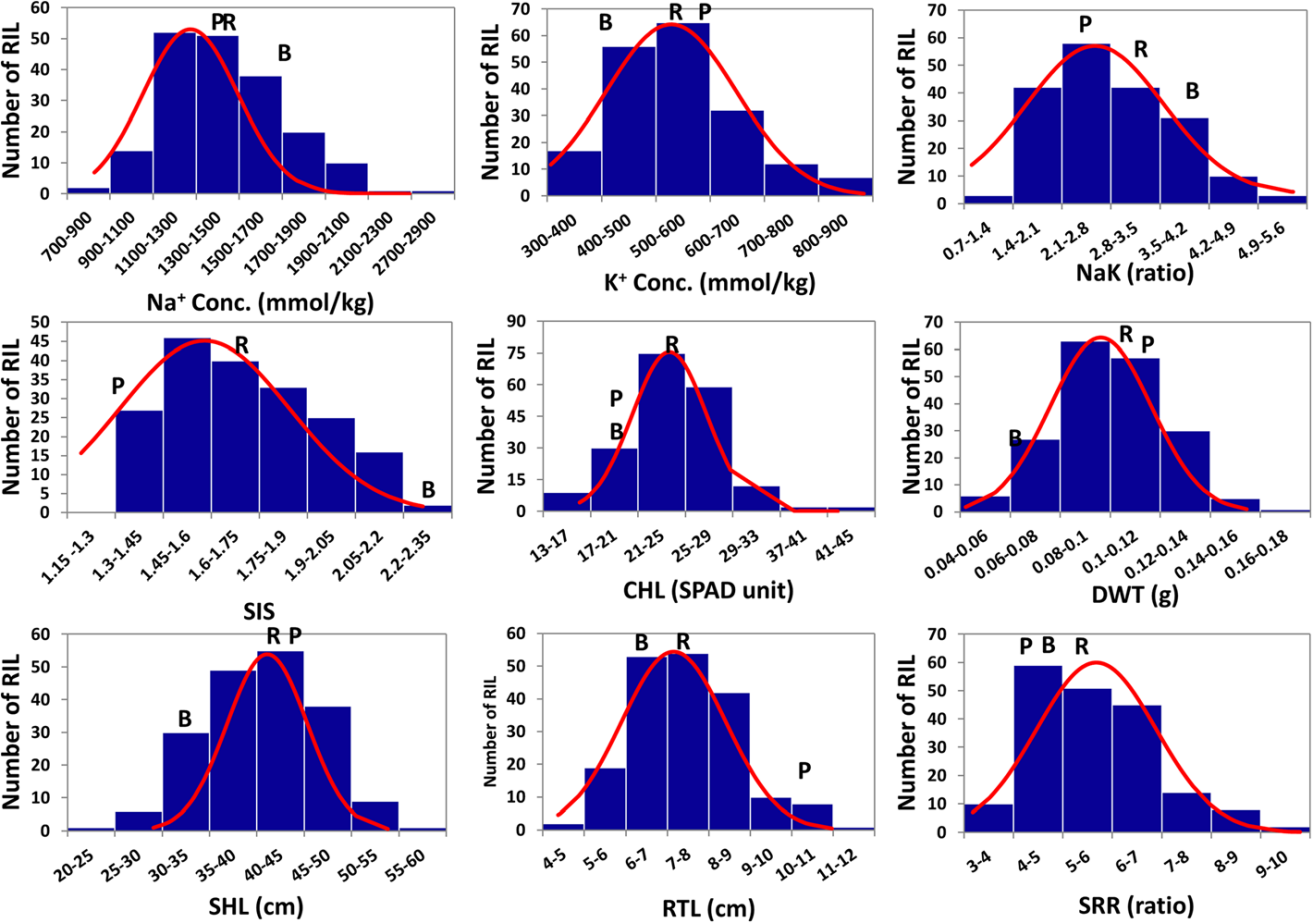
Frequency distribution of Bengal/Pokkali F_6_ RIL population for traits related to seedling salinity tolerance. Na^+^ Conc., Na^+^ concentration; K^+^ conc., K^+^ concentration; NaK, Na^+^/K^+^ ratio; SIS, log transformed salt injury score; CHL, chlorophyll content measured by SPAD-502 unit; DWT, dry weight; SHL, shoot length; RTL, root length; SRR, Shoot length to root length ratio

The parents and RIL population were evaluated under salt stress for salt injury score (SIS), chlorophyll content (CHL), shoot length (SHL), root length (RTL), shoot length to root length ratio (SRR), dry shoot weight (DWT), shoot Na^+^ and K^+^ concentrations, and Na^+^/K ^+^ ratio (NaK ratio). At 12dSm^-1^ salt stress, the RILs and parents showed varying levels of tolerance. Bengal and Pokkali showed significant contrasting response in SIS, SHL, RTL, DWT, and NaK ratio (Table 1). However, the differences in CHL, SRR, Na^+^ and K^+^ concentrations, were not statistically significant between parents. Pokkali showed consistently lower SIS, Na^+^ concentration, NaK ratio, and higher K^+^ concentration than Bengal. Among the RILs, all traits showed significant genotypic differences (p <0.0001), indicating a wide range of variation. The RIL population had a mean value between the parental means for all traits except in CHL and SRR. Pokkali had an average SIS of 3; Bengal had 8.4, while the RILs had a mean SIS of 4.7. The RIL population had a mean Na^+^ accumulation of 1430 mmolkg^-1^ in shoot, which is much lower than Bengal (1700 mmolkg^-1^), and marginally higher than Pokkali (1424 mmolkg^-1^). In contrast, the mean K^+^ accumulation was highest in Pokkali (591 mmolkg^-1^), followed by RILs (547 mmolkg^-1^) and lowest in Bengal (420 mmolkg^-1^). The RIL population had mean chlorophyll content greater than either parent. As indicated in the frequency distribution (Fig. 1) and the range of RIL values for each trait (Table 1), several lines were phenotypically superior to the parents. There were many transgressive segregants with much lower Na^+^ than Bengal, lower NaK ratio and SHL and higher CHL, DWT, RTL and SRR than Bengal or Pokkali. Similarly, some lines accumulated twice the K^+^ concentration of Pokkali. But there was no line that showed higher tolerance than Pokkali as judged by SIS (Fig. 1). There was wide variation for heritability values for traits. Heritabilities for Na^+^, K^+^ concentrations, and SHL were 0.98, 0.95, and 90, respectively. In contrast, NaK ratio, SIS, CHL, RTL, and SRR had moderate heritability of 0.24–0.63 while DWT has very low heritability.

**Table 1:** Phenotypic response of parents and F_6_ RIL population for traits related to salt tolerance at seedling stage

Trait Name	Bengal Mean	Pokkali Mean^β^	RIL Mean	Std. Dev.	RIL Range	RIL Pr > F^§^	Heritability^¥^
Na^+^ (mmolkg^-1^)	1700.00	1424.3^ns^	1430.7	246.24	861.97–2733.35	<0.0001	0.98
K^+^ (mmolkg^-1^)	420.00	591^ns^	547.3	107.59	335.99–884.18	<0.0001	0.95
NaK (ratio)	4.07	2.38**	2.8	0.56	1.25–5.32	<0.0001	0.24
SIS	8.40	3.00***	4.7	0.72	3.00–8.73	<0.0001	0.44
CHL (SPAD unit)	20.56	19.54^ns^	24.2	4.25	13.72–43.67	<0.0001	0.45
SHL (cm)	32.07	44.52***	40.7	3.21	22.60–59.73	<0.0001	0.90
RTL (cm)	6.73	10.08**	7.4	0.64	4.67–11.27	<0.0001	0.61
DWT (g)	0.06	0.11*	0.1	0.01	0.04–0.16	<0.0001	0.01
SRR (ratio)	4.98	4.53^ns^	5.6	0.53	3.08–9.79	<0.0001	0.63

Na^+^: shoot sodium concentration, K^+^: shoot potassium concentration, NaK: ratio of the shoot sodium and shoot potassium content, SIS: salt injury score, CHL: chlorophyll content, SHL: shoot length, RTL: root length, DWT: shoot dry weight, SRR: shoot length to root length ratio

^β^Significant differences between Bengal and Pokkali, ^ns^no significant differences, *significant at 0.05 probability level, **significant at 0.01 probability level, ***significant at 0.001 probability level

^§^Genotypic differences among RIL

^¥^Broad sense heritability computed on family mean basis

### Correlation of Traits

Correlations among all traits (Table 2) revealed that SIS was highly significant and positively correlated to Na^+^ concentration and NaK ratio. The SIS was highly significant and negatively correlated to CHL, SHL, RTL, DWT and SRR, indicating the negative effect of salt stress on the overall growth and photosynthetic capability of plants. On the other hand, K^+^ concentration was positively correlated to Na^+^ concentration, SHL, CHL, DWT, and SRR but negatively correlated to NaK ratio, SIS, and RTL. The relationships between traits in RIL population were consistent to the correlation of traits observed among the 30 US rice genotypes (De Leon et al. 2015), thus indicating reliability and reproducibility of our salt tolerance screening.

**Table 2:** Pearson correlation matrix of traits measured in response to salt stress at 12dSm^-1^ in Bengal/Pokkali F_6_ RIL population at seedling stage

	Na^+^	K^+^	NaK	SIS	CHL	SHL	RTL	DWT	SRR
Na^+^	1								
K^+^	0.1271**	1							
NaK	0.594***	-0.649***	1						
SIS	0.337***	-0.129**	0.337***	1					
CHL	-0.128**	0.092*	-0.157***	-0.214***	1				
SHL	0.039	0.253***	-0.151***	-0.236***	0.221***	1			
RTL	0.057	-0.105*	0.095*	-0.109**	0.059	0.204***	1		
DWT	0.006	0.144***	-0.099*	-0.475***	0.177***	0.539***	0.279***	1	
SRR	-0.024	0.277***	-0.195***	-0.099*	0.111**	0.593***	-0.638***	0.173*	1

Na^+^: shoot sodium concentration, K^+^: shoot potassium concentration, NaK: ratio of the shoot sodium and shoot potassium content, SIS: salt injury score, CHL: chlorophyll content, SHL: shoot length, RTL: root length, DWT: dry weight, SRR: shoot length to root length ratio

*significant at 0.05 probability level, **significant at 0.01 probability level, ***significant at 0.001 probability level

### Linkage Mapping

**Fig. 2:**
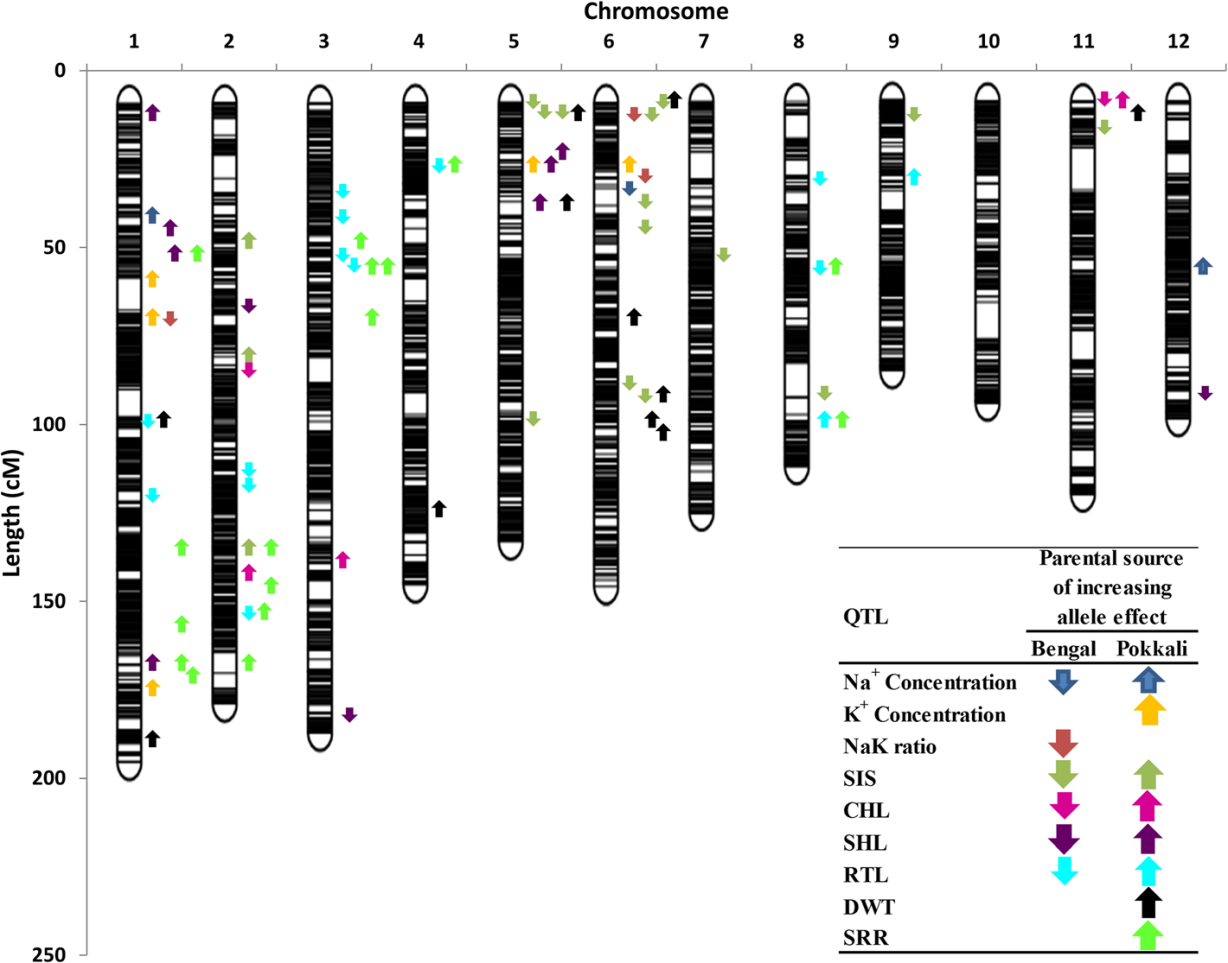
Molecular genetic map showing the positions of QTLs for nine traits investigated under salt stress. Linkage and QTL mapping were implemented in ICIM QTL Mapping 4.0 using 9303 GBS-SNP markers in 187 Bengal/Pokkali F_6_ RILs. Chromosome regions that are dark indicate the saturation of markers while regions that are white indicate the absence of marker placed in those segments. Genetic distance in centimorgan was determined by Kosambi map function. Each arrow represents a single QTL for a particular trait

GBS generated a total of 33,987 SNP markers which were furtherly filtered for polymorphic markers and for markers with less than 10 % missing data across the population. A total of 9303 SNPs markers were retained and used in the linkage map construction (Fig. 2, Additional file 1: Table S1). On the average, about 775 SNP markers were placed per chromosome (Table 3). The final linkage map had a total length of 1650 cM with 2817 recombination sites. The average distance between adjacent markers was 0.59 cM or 39,798 bp, with maximum resolution of 0.27 cM. The average marker density was 5.6 SNP markers per cM or 3.3 SNP markers per recombination point. The map was saturated with SNP markers across all chromosomes. However, twenty large gaps were observed on chromosomes 1, 2, 3, 4, 6, 7, 8, 10, 11, and 12 that ranged between 5 cM to 13 cM. With 9303 SNP markers, the linkage map had a physical to genetic map length ratio of 225 Kb/cM.

**Table 3:** Summary distribution, coverage, and intervals of SNP markers in the Bengal/Pokkali RIL linkage map

Chromosome	No. of SNP markers used	Chromosome length coverage (Mb)	Genetic length (cM)	No. of recombination points	No. of SNP markers/cM	No. of SNP markers/unique position	Minimum interval (cM)	Maximum interval (cM)	Average Interval (cM)	No. of Gaps >5 cM
1	1245	43,237,333	199.8	363	6.2	3.4	0.27	9.98	0.55	2
2	1001	35,875,736	182.9	324	5.5	3.1	0.27	7.19	0.56	2
3	1068	36,405,799	191.2	320	5.6	3.3	0.28	8.01	0.60	2
4	822	35,501,387	148.1	244	5.6	3.4	0.27	6.88	0.60	2
5	780	29,507,277	135.6	243	5.8	3.2	0.27	4.35	0.56	0
6	842	30,869,147	148.6	258	5.7	3.3	0.27	5.33	0.57	1
7	736	29,582,943	127.4	225	5.8	3.3	0.27	8.7	0.57	1
8	471	28,399,689	113.9	162	4.1	2.9	0.27	10.57	0.70	3
9	584	22,779,506	85.9	164	6.8	3.6	0.27	6.49	0.52	1
10	517	23,117,196	95.2	149	5.4	3.5	0.28	11.55	0.64	1
11	622	28,973,227	121.8	187	5.1	3.3	0.28	13.09	0.65	3
12	615	27,488,377	99.9	178	6.2	3.5	0.27	6.75	0.56	2
Total	9303	371,737,617	1650.2	2817	67.7	39.7	3.27	98.89	7.08	20
Average^a^	775.3	30,978,134.75	137.5	234.8	5.6	3.3	0.27	8.24	0.59	1.7

^a^Average value per chromosome

### Identification of Additive and Di-genic Epistatic QTLs for Traits Related to Salinity Tolerance

To detect novel additive and epistatic QTLs for traits related to salinity tolerance, the phenotype and GBS data were used in interval mapping (IM) and inclusive composite interval mapping (ICIM) methods.

#### QTLs for Shoot Na^+^ Concentration

The IM and ICIM methods consistently detected three additive QTLs for shoot Na^+^ concentration (Table 4). The QTLs were located on chromosomes 2, 6, and 12. Each additive QTL explained at least 5.5 % of the phenotypic variation. Pokkali alleles of *qNa2.7* and *qNa12.18* had increasing effect while for *qNa6.5* Bengal allele had the increasing effect. Interval mapping of epistatic QTLs detected seven pairs of QTLs with significant contribution to the variation in Na^+^ concentration (Table 5). Four of the seven pairs of epistatic QTLs had large effects (PVE = 11–16 %) while the other three pairs had small effects (PVE = 8–9 %). Nine interacting QTLs with increasing effect were from Bengal and five were from Pokkali. None of the additive QTLs co-localized with epistatic QTLs.

**Table 4:** Additive QTLs for traits related to seedling-stage salt tolerance in Bengal/Pokkali F_6_ RIL population identified by IM and ICIM methods

Phenotype	QTL	Chr^a^	Position (cM)	Left Marker	Right Marker	QTL Interval Size (bp)	LOD	PVE (%)	Additive Effect	Parental Source of Increasing Allele Effect^b^	No. of genes in QTL interval
Na^+^ concentration-IM	*qNa2.7*	2	48	S2_7769844	S2_7939496	169,652	2.2969	5.55	-66.59	P	24
	*qNa6.5*	6	34	S6_5269698	S6_5533752	264,054	2.399	5.97	69.15	B	34
	*qNa12.18*	12	60	S12_18687038	S12_18741493	54,455	2.2496	5.51	-66.36	P	5
Na^+^ concentration-ICIM	*qNa2.7*	2	48	S2_7769844	S2_7939496	169,652	2.2969	5.55	-66.59	P	24
	*qNa6.5*	6	34	S6_5269698	S6_5533752	264,054	2.399	5.97	69.15	B	34
	*qNa12.18*	12	60	S12_18687038	S12_18741493	54,455	2.2496	5.51	-66.36	P	5
K^+^ concentration-IM	*qK1.8*	1	63	S1_8656025	S1_8901503	245,478	5.7933	13.65	-46.34	P	33
	*qK1.11*	1	71	S1_11529325	S1_11581799	52,474	5.9263	13.66	-45.13	P	6
	*qK1.38*	1	173	S1_38794029	S1_39047133	253,104	3.5096	8.30	-33.32	P	40
	*qK5.4*	5	31	S5_4699921	S5_5326365	626,444	2.2496	5.51	-27.00	P	86
	*qK6.4*	6	31	S6_4890290	S6_5269698	379,408	3.333	8.21	-32.92	P	61
K^+^ concentration-ICIM	*qK1.11*	1	71	S1_11529325	S1_11581799	52,474	7.7414	16.08	-48.95	P	6
	*qK1.38*	1	173	S1_38794029	S1_39047133	253,104	5.3793	10.71	-37.86	P	40
NaK ratio-IM	*qNaK1.11*	1	71	S1_11529325	S1_11581799	52,474	4.154	9.83	0.29	B	6
	*qNaK6.2*	6	15	S6_2927160	S6_2962502	35,342	3.5777	8.46	0.26	B	7
	*qNaK6.5*	6	33	S6_5269698	S6_5533752	264,054	5.1164	13.21	0.32	B	34
NaK ratio-ICIM	*qNaK1.11*	1	71	S1_11529325	S1_11581799	52,474	2.6375	5.66	0.22	B	6
	*qNaK6.5*	6	33	S6_5269698	S6_5533752	264,054	3.7097	8.85	0.26	B	34
Salt injury score-IM	*qSIS2.8*	2	50	S2_8730258	S2_8927908	197,650	3.5375	8.58	-0.06	P	25
	*qSIS2.19*	2	81	S2_19331684	S2_19454952	123,268	3.2133	7.66	-0.06	P	14
	*qSIS2.28*	2	131	S2_28239596	S2_28274467	34,871	2.6449	6.37	-0.05	P	8
	*qSIS5.03*	5	1	S5_312457	S5_329699	17,242	2.8257	6.74	0.06	B	4
	*qSIS5.1a*	5	12	S5_1686924	S5_1707475	20,551	2.8266	6.76	0.06	B	5
	*qSIS5.24*	5	106	S5_24057323	S5_24281632	224,309	3.1266	7.51	0.06	B	39
	*qSIS6.2*	6	15	S6_2927160	S6_2962502	35,342	2.0824	5.04	0.05	B	7
	*qSIS6.5*	6	37	S6_5848568	S6_5905669	57,101	3.0401	7.23	0.06	B	11
	*qSIS6.7*	6	48	S6_7646442	S6_7661883	15,441	3.1238	7.41	0.06	B	3
	*qSIS6.20*	6	90	S6_20929261	S6_20929283	22	3.9605	9.44	0.07	B	1
	*qSIS11.2*	11	18	S11_2838776	S11_3716306	877,530	2.664	8.36	0.06	B	136
Salt injury score-ICIM	*qSIS5.1b*	5	11	S5_1441967	S5_1454837	12,870	9.7068	13.33	0.08	B	2
	*qSIS6.2b*	6	9	S6_2123411	S6_2242943	119,532	3.5933	4.46	0.05	B	23
	*qSIS6.21*	6	92	S6_21253244	S6_21256132	2888	6.9204	9.11	0.07	B	1
	*qSIS7.14*	7	57	S7_14598897	S7_14625841	26,944	3.6209	4.50	0.05	B	7
	*qSIS8.24*	8	93	S8_24763939	S8_25110888	346,949	2.6235	3.28	0.04	B	47
	*qSIS9.8*	9	13	S9_8608506	S9_9070610	462,104	7.09	9.19	0.07	B	51
	*qSIS11.2*	11	21	S11_2838776	S11_3716306	877,530	2.336	3.53	0.04	B	136
Chlorophyll content-IM	*qCHL11.1*	11	5	S11_1086712	S11_1293020	206,308	2.1922	5.41	-1.00	P	34
	*qCHL11.2*	11	14	S11_2666525	S11_2724222	57,697	2.0172	4.86	-0.95	P	7
Chlorophyll content-ICIM	*qCHL2.20*	2	86	S2_20258450	S2_20346560	88,110	3.6938	7.44	1.18	B	7
	*qCHL2.30*	2	143	S2_30353435	S2_30402468	49,033	2.3418	4.69	-0.94	P	7
	*qCHL3.26*	3	136	S3_26705619	S3_26709038	3419	3.2263	6.42	-1.10	P	1
Shoot length-IM	*qSHL1.1*	1	11	S1_1708228	S1_1747144	38,916	2.0367	5.03	-1.42	P	7
	*qSHL1.7a*	1	48	S1_7259818	S1_7296346	36,528	3.9307	9.26	-1.95	P	7
	*qSHL1.38*	1	168	S1_38286772	S1_38611845	325,073	25.3529	48.03	-4.43	P	52
	*qSHL3.34*	3	185	S3_34720589	S3_35060080	339,491	2.361	5.65	1.54	B	69
	*qSHL5.4*	5	29	S5_4565557	S5_4699921	134,364	2.3239	5.64	-1.52	P	23
	*qSHL5.6*	5	44	S5_6356744	S5_6433933	77,189	2.0324	4.96	-1.41	P	13
Shoot length-ICIM	*qSHL1.7b*	1	50	S1_7520182	S1_7569628	49,446	6.2678	5.86	-1.57	P	5
	*qSHL1.38*	1	168	S1_38286772	S1_38611845	325,073	36.9075	51.64	-4.59	P	52
	*qSHL2.18*	2	77	S2_18806154	S2_18937362	131,208	3.0049	2.71	1.04	B	25
	*qSHL3.34*	3	185	S3_34720589	S3_35060080	339,491	4.3994	3.96	1.29	B	69
	*qSHL5.3*	5	25	S5_3353753	S5_3506138	152,385	7.0797	6.79	-1.66	P	21
	*qSHL12.25*	12	93	S12_25709174	S12_25887173	177,999	2.246	2.05	0.91	B	30
Root length-IM	*qRTL1.26*	1	121	S1_26421289	S1_26447134	25,845	2.7346	6.52	0.32	B	6
	*qRTL2.24*	2	114	S2_24961302	S2_24961342	40	4.1447	9.72	0.39	B	0
	*qRTL2.26*	2	120	S2_26028043	S2_26070191	42,148	4.2053	9.91	0.39	B	9
	*qRTL2.33*	2	160	S2_33573567	S2_33614297	40,730	3.9359	9.50	0.39	B	7
	*qRTL3.6*	3	36	S3_6011601	S3_6027452	15,851	3.4683	8.23	0.36	B	2
	*qRTL3.7*	3	44	S3_7130220	S3_7209963	79,743	4.4685	10.70	0.41	B	15
	*qRTL3.10*	3	57	S3_10116591	S3_10132745	16,154	5.0358	11.99	0.43	B	2
	*qRTL4.10*	4	24	S4_10625625	S4_10726368	100,743	2.0131	4.88	0.28	B	14
	*qRTL8.4*	8	37	S8_4558562	S8_4858127	299,565	2.121	5.34	0.36	B	41
	*qRTL8.19*	8	59	S8_19884635	S8_19898432	13,797	3.272	7.75	0.41	B	2
	*qRTL8.27*	8	109	S8_27238050	S8_27304101	66,051	2.1036	5.13	-0.28	P	9
	*qRTL9.14*	9	39	S9_14960521	S9_14976723	16,202	2.6572	6.45	-0.36	P	3
Root length-ICIM	*qRTL1.22*	1	102	S1_22666852	S1_22677418	10,566	2.2657	3.54	0.23	B	2
	*qRTL1.26*	1	121	S1_26421289	S1_26447134	25,845	2.1764	3.41	0.23	B	6
	*qRTL3.9*	3	56	S3_9853159	S3_9891061	37,902	4.2907	7.59	0.34	B	7
Dry weight-IM	*qDWT1.21*	1	97	S1_21707357	S1_21733437	26,080	2.3413	5.60	-0.01	P	6
	*qDWT4.32*	4	126	S4_32367131	S4_32367159	28	2.3915	5.73	-0.01	P	1
	*qDWT5.2*	5	15	S5_2116055	S5_2167880	51,825	4.6334	10.80	-0.01	P	4
	*qDWT5.4*	5	29	S5_4565557	S5_4699921	134,364	6.5783	15.04	-0.01	P	23
	*qDWT5.5*	5	42	S5_5997340	S5_6196044	198,704	6.5368	15.47	-0.01	P	32
	*qDWT6.13*	6	72	S6_13046472	S6_13097774	51,302	2.0119	5.16	0.00	P	10
	*qDWT6.20*	6	90	S6_20929261	S6_20929283	22	3.7538	8.95	-0.01	P	1
	*qDWT6.23*	6	102	S6_23812023	S6_24039384	227,361	3.7054	8.91	-0.01	P	32
	*qDWT11.2*	11	10	S11_2379158	S11_2402109	22,951	2.4389	6.03	-0.01	P	3
Dry weight-ICIM	*qDWT1.40*	1	185	S1_40372283	S1_40412316	40,033	2.0722	3.13	0.00	P	6
	*qDWT4.32*	4	126	S4_32367131	S4_32367159	28	3.6566	5.93	-0.01	P	1
	*qDWT5.4*	5	29	S5_4565557	S5_4699921	134,364	7.5727	12.98	-0.01	P	23
	*qDWT6.06*	6	3	S6_692773	S6_782975	90,202	3.7128	6.02	-0.01	P	13
	*qDWT6.24*	6	104	S6_24107596	S6_24228831	121,235	4.4604	7.46	-0.01	P	19
Shoot-root ratio-IM	*qSRR1.7*	1	50	S1_7520182	S1_7569628	49,446	3.7944	9.09	-0.38	P	5
	*qSRR1.29*	1	135	S1_29561423	S1_29568978	7555	3.1151	7.42	-0.33	P	2
	*qSRR1.36*	1	159	S1_36158467	S1_36189206	30,739	5.8447	13.42	-0.45	P	5
	*qSRR1.382*	1	170	S1_38286772	S1_38611845	325,073	10.3107	23.01	-0.59	P	52
	*qSRR2.28*	2	133	S2_28317911	S2_28375704	57,793	4.7052	10.96	-0.41	P	7
	*qSRR2.31*	2	146	S2_31037977	S2_31043939	5962	3.2045	7.62	-0.34	P	1
	*qSRR2.33*	2	160	S2_33573567	S2_33614297	40,730	4.1813	9.90	-0.39	P	7
	*qSRR2.34*	2	168	S2_34660774	S2_35085922	425,148	2.9367	7.37	-0.33	P	68
	*qSRR3.8*	3	49	S3_8327882	S3_8353264	25,382	2.6453	6.32	-0.31	P	6
	*qSRR3.10*	3	57	S3_10116591	S3_10132745	16,154	2.6902	6.58	-0.31	P	2
	*qSRR3.11*	3	70	S3_11848358	S3_11865689	17,331	2.4751	5.93	-0.30	P	1
	*qSRR4.10*	4	24	S4_10625625	S4_10726368	100,743	2.438	5.91	-0.30	P	14
	*qSRR8.19*	8	59	S8_19884635	S8_19898432	13,797	2.3793	5.70	-0.35	P	2
Shoot root ratio-ICIM	*qSRR1.7*	1	50	S1_7520182	S1_7569628	49,446	6.9282	8.73	-0.37	P	5
	*qSRR1.386*	1	171	S1_38636497	S1_38768787	132,290	15.6449	22.43	-0.59	P	22
	*qSRR2.33*	2	160	S2_33573567	S2_33614297	40,730	8.5304	10.92	-0.41	P	7
	*qSRR3.9*	3	56	S3_9853159	S3_9891061	37,902	4.3278	5.25	-0.28	P	7
	*qSRR8.26*	8	107	S8_26716230	S8_26744324	28,094	2.533	3.01	0.21	B	5

^a^ Chromosome where the QTL was located. ^b^Parental source of increasing allele effect was either Pokkali (P) or Bengal (B)

**Table 5:** Di-genic epistatic QTLs for traits related to salt tolerance at seedling stage in Bengal/Pokkali F6 RIL population identified by interval mapping

Phenotype	QTL1	Chr.1	Position1	LeftMarker1	RightMarker1	QTL2	Chr.2	Position2 (cM)	LeftMarker2	RightMarker2	LOD	PVE(%)	Add1	Add2	Add x Add
Na^+^ concentration	*qNa4.25*	4	90	S4_25549517	S4_26622324	*qNa4.29*	4	110	S4_29966056	S4_29968457	3.08	11.02	57.92	-21.10	-102.91
	*qNa3.26*	3	135	S3_26536286	S3_26542118	*qNa5.008*	5	0	S5_87749	S5_96410	3.24	7.90	-13.88	-0.72	78.59
	*qNa1.12*	1	75	S1_12583448	S1_12685974	*qNa6.2*	6	10	S6_2266152	S6_2272501	3.34	9.09	43.52	-14.46	90.61
	*qNa6.17*	6	80	S6_17631626	S6_17780076	*qNa6.19*	6	85	S6_19446057	S6_19585327	3.10	15.64	106.90	-89.69	-208.67
	*qNa6.4a*	6	25	S6_4631489	S6_4771954	*qNa10.21*	10	85	S10_21364298	S10_21407693	3.83	13.99	86.22	46.66	88.20
	*qNa6.4b*	6	30	S6_4890290	S6_5269698	*qNa11.1*	11	5	S11_1086712	S11_1293020	3.26	13.91	58.04	39.35	80.06
	*qNa3.2*	3	15	S3_2171559	S3_2250307	*qNa11.23*	11	100	S11_23611942	S11_23708208	3.16	8.69	5.27	10.52	82.87
K^+^ concentration	*qK1.7*	1	60	S1_7778029	S1_8656025	*qK2.3*	2	15	S2_3207423	S2_3207477	3.50	21.42	-44.62	-22.75	36.16
	*qK2.25*	2	115	S2_25166702	S2_25192275	*qK3.22*	3	115	S3_22976923	S3_23020366	3.04	8.02	5.08	-5.45	31.47
	*qK1.40*	1	190	S1_40584495	S1_40894634	*qK7.19*	7	70	S7_19334046	S7_19406235	3.14	9.07	-12.92	1.97	-32.60
	*qK1.7*	1	50	S1_7520182	S1_7569628	*qK12.17*	12	55	S12_17065005	S12_17195754	3.23	9.03	-13.81	8.04	-32.71
	*qK11.19*	11	70	S11_19222100	S11_19245359	*qK12.18*	12	60	S12_18687038	S12_18741493	3.63	10.31	16.55	-3.15	-34.00
NaK ratio	*qNaK1.42*	1	195	S1_42138516	S1_42310908	*qNaK3.21*	3	110	S3_21445493	S3_21628785	3.08	8.71	0.10	0.02	-0.24
	*qNaK6.30*	6	145	S6_30296317	S6_30370989	*qNaK8.2*	8	20	S8_2341829	S8_2949528	3.09	8.68	0.07	-0.13	0.26
	*qNaK6.4a*	6	25	S6_4631489	S6_4771954	*qNaK10.213*	10	85	S10_21364298	S10_21407693	3.58	17.65	0.34	0.12	0.26
	*qNaK7.22*	7	90	S7_22936622	S7_22936634	*qNaK10.217*	10	90	S10_21749293	S10_21786307	3.47	8.73	-0.03	0.03	-0.26
	*qNaK5.16*	5	65	S5_16290294	S5_16307102	*qNaK11.2*	11	15	S11_2838776	S11_3716306	3.55	10.80	-0.05	0.09	-0.26
	*qNaK3.2*	3	20	S3_2776106	S3_2780171	*qNaK11.24*	11	105	S11_24319577	S11_24335733	3.46	9.89	0.00	-0.12	0.26
	*qNaK1.5*	1	35	S1_5501756	S1_5792183	*qNaK12.19*	12	65	S12_19926993	S12_20016304	3.01	8.66	0.06	-0.08	0.24
Salt injury score	*qSIS6.2a*	6	15	S6_2927160	S6_2962502	*qSIS6.30*	6	145	S6_30296317	S6_30370989	3.82	15.04	0.04	0.04	0.07
	*qSIS5.18*	5	80	S5_18942631	S5_18997491	*qSIS9.9*	9	15	S9_9351804	S9_9857266	3.16	12.06	0.05	0.05	0.06
	*qSIS3.10*	3	65	S3_10992290	S3_11053944	*qSIS10.2*	10	5	S10_2799960	S10_2837737	4.11	11.59	0.01	0.04	0.07
	*qSIS2.20*	2	85	S2_20153436	S2_20182321	*qSIS10.11*	10	25	S10_11045261	S10_11244588	3.07	14.67	-0.07	0.02	-0.06
	*qSIS3.11*	3	70	S3_11848358	S3_11865689	*qSIS12.2*	12	15	S12_2315570	S12_2397199	3.33	7.96	0.00	0.00	0.06
Chlorophyll	*qCHL1.20*	1	90	S1_20242882	S1_21276489	*qCHL1.21*	1	95	S1_21276489	S1_21352851	7.27	29.81	-4.09	4.17	-5.07
content	*qCHL3.17*	3	105	S3_17083355	S3_17143997	*qCHL3.21*	3	110	S3_21445493	S3_21628785	4.59	28.05	4.16	-4.42	-4.73
	*qCHL3.21*	3	110	S3_21445493	S3_21628785	*qCHL7.7*	7	50	S7_7781645	S7_7839200	3.31	8.45	-0.29	-0.20	-1.23
	*qCHL8.23*	8	90	S8_23657286	S8_24738259	*qCHL8.24*	8	95	S8_24763939	S8_25110888	5.38	35.72	-4.73	5.06	-3.76
	*qCHL9.12*	9	25	S9_12217170	S9_12366675	*qCHL9.12*	9	30	S9_12915373	S9_14359383	3.89	34.51	-4.77	4.51	-4.09
	*qCHL2.5*	2	40	S2_5800279	S2_5848583	*qCHL9.18*	9	60	S9_18667894	S9_18669560	3.04	7.74	-0.17	-0.44	-1.20
	*qCHL10.18*	10	65	S10_18819950	S10_19941928	*qCHL10.18*	10	70	S10_18819950	S10_19941928	5.68	34.16	-4.44	4.32	-4.33
	*qCHL7.27*	7	110	S7_27772814	S7_27803479	*qCHL10.21*	10	90	S10_21749293	S10_21786307	3.52	13.31	1.14	0.91	1.28
	*qCHL11.4*	11	35	S11_4854309	S11_4863888	*qCHL11.6*	11	40	S11_6970703	S11_7012013	3.97	11.76	-1.18	1.25	-2.15
	*qCHL3.4*	3	25	S3_4116916	S3_4311471	*qCHL11.24*	11	105	S11_24319577	S11_24335733	3.68	11.82	0.28	-0.53	-1.30
Shoot length	*qSHL2.1*	2	10	S2_1653448	S2_2064517	*qSHL2.5*	2	40	S2_5800279	S2_5848583	3.84	9.77	-0.12	-0.48	-2.02
	*qSHL4.25*	4	95	S4_25549517	S4_26622324	*qSHL5.008*	5	0	S5_87749	S5_96410	4.32	10.89	-0.16	-0.22	-2.07
	*qSHL4.27*	4	100	S4_27678052	S4_27715999	*qSHL8.1*	8	15	S8_1995144	S8_2005542	3.81	10.15	-0.23	0.09	1.97
	*qSHL2.32*	2	155	S2_32339457	S2_32429009	*qSHL9.12*	9	25	S9_12217170	S9_12366675	3.67	11.31	0.82	-1.54	2.23
	*qSHL1.28*	1	130	S1_28157998	S1_28247178	*qSHL9.19*	9	65	S9_19628929	S9_19696641	3.09	11.19	-0.72	-0.54	1.75
	*qSHL2.34*	2	165	S2_34519074	S2_34545438	*qSHL10.20*	10	80	S10_20682624	S10_20733813	3.34	9.36	-0.15	0.46	1.83
	*qSHL4.32*	4	130	S4_32867449	S4_33074444	*qSHL10.21*	10	90	S10_21749293	S10_21786307	3.47	10.80	-1.40	-0.05	-1.86
Root length	*qRTL1.32*	1	145	S1_32327040	S1_32418346	*qRTL3.10*	3	65	S3_10992290	S3_11053944	4.80	17.47	-0.03	0.30	0.41
	*qRTL4.16*	4	35	S4_16669714	S4_16706375	*qRTL6.25*	6	115	S6_25296416	S6_25363541	3.79	12.10	0.19	-0.12	0.37
	*qRTL3.28*	3	145	S3_28513488	S3_29240341	*qRTL8.23*	8	90	S8_23657286	S8_24738259	3.26	9.34	-0.11	-0.05	-0.38
	*qRTL6.15*	6	75	S6_15734275	S6_15881397	*qRTL9.16*	9	50	S9_16775205	S9_16882286	3.02	11.22	0.15	-0.29	0.36
	*qRTL4.33*	4	135	S4_33557881	S4_33861248	*qRTL10.19*	10	75	S10_19941928	S10_20082337	4.15	10.32	-0.01	0.02	-0.41
Dry weight	*qDWT3.17*	3	105	S3_17083355	S3_17143997	*qDWT6.7*	6	50	S6_7662391	S6_7749349	3.20	10.64	0.00	0.00	-0.01
	*qDWT6.4*	6	30	S6_4890290	S6_5269698	*qDWT6.30*	6	145	S6_30296317	S6_30370989	3.06	12.61	0.00	-0.01	-0.01
	*qDWT7.1*	7	5	S7_1021298	S7_1051320	*qDWT7.27*	7	110	S7_27772814	S7_27803479	3.43	10.08	0.00	0.00	0.01
	*qDWT5.2*	5	20	S5_2483311	S5_2495045	*qDWT10.16*	10	50	S10_16848745	S10_16898283	3.30	16.39	-0.01	0.00	-0.01
	*qDWT4.16*	4	35	S4_16669714	S4_16706375	*qDWT10.19*	10	75	S10_19941928	S10_20082337	3.34	9.61	0.00	0.00	0.01
	*qDWT3.5*	3	35	S3_5859095	S3_5904925	*qDWT12.09*	12	10	S12_977852	S12_1386213	3.80	12.95	0.00	0.00	-0.01
	*qSRR2.1*	2	5	S2_1103758	S2_1653448	*qSRR4.27*	4	100	S4_27678052	S4_27715999	3.47	11.08	0.00	-0.19	0.35
Shoot-root ratio	*qSRR5.2*	5	15	S5_2116055	S5_2167880	*qSRR5.5*	5	40	S5_5798670	S5_5909747	3.85	9.93	0.12	-0.07	-0.39
	*qSRR2.3*	2	25	S2_3978527	S2_4234638	*qSRR9.14*	9	40	S9_14976723	S9_15092089	3.20	10.65	-0.14	0.24	-0.39
	*qSRR1.20*	1	90	S1_20242882	S1_21276489	*qSRR9.21*	9	75	S9_21030508	S9_21083576	3.09	10.97	-0.11	-0.13	0.37
	*qSRR4.18*	4	45	S4_18779374	S4_18826971	*qSRR10.001*	10	0	S10_103050	S10_160013	3.18	9.97	-0.13	-0.16	0.34

#### QTLs for Shoot K^+^ Concentration

The IM method detected five additive QTLs (*qK1.8, qK1.11, qK1.38, qK5.4,* and *qK6.4*) for shoot K^+^ concentration. The *qK1.8* and *qK1.11* were large-effect QTLs, each accounting for at least 13 % of the variation for shoot K^+^. The other three QTLs had small effects (5–8 % PVE) and were located on chromosomes 1, 5, and 6. The *qK1.11 and qK1.38* were also detected by ICIM with LOD values of 7.7 and 5.4, respectively. Both *qK1.11* and *qK1.38* were large-effect QTLs in ICIM method with PVE of 16 and 10 %. In contrast, *qK1.8, qK5.4,* and *qK6.4* were not detected in ICIM. All additive QTLs for K^+^ concentration had increasing effects that originated from Pokkali, indicating the importance of Pokkali alleles for increased uptake of K^+^ in the leaves. Five pairs of epistatic QTLs were detected for K^+^ concentration. The *qK1.7* and the *qK2.3* pair had a PVE of 21 % and LOD score of 3.5, with Pokkali allele contributing for increased K^+^ accumulation. The *qK1.7* also interacted with *qK12.17* and accounted for 9 % of the variation in K^+^ accumulation. Additionally, *qK11.19* and *qK12.18* pair had a PVE of 10 % while the remaining two pairs accounted for 9 % of the phenotypic variation. Six and four interacting QTLs with increasing effects involved Pokkali and Bengal alleles, respectively. All additive QTL positions were independent of epistatic QTLs.

#### QTLs for NaK Ratio

For NaK ratio, three additive QTLs (*qNaK1.11, qNaK6.2, qNaK6.5*) were significant in IM method but only two of the additive QTLs (*qNaK1.11, qNaK6.5*) were detected in ICIM. The *qNaK 6.5* explained 13 % of the phenotypic variation while *qNaK6.2* and *qNaK1.11* were small-effect QTLs. All NaK ratio QTLs had increasing effects due to Bengal alleles. Of the seven pairs of epistatic QTLs, two pairs were large-effect QTLs (PVE = 11 and 18 %) and five pairs were minor QTLs with PVEs lower than 9 %. There was no epistatic QTL found in the same chromosome intervals of additive QTLs for NaK ratio, K^+^, or Na^+^ concentrations. Most of the QTL alleles with increasing effects were from Bengal. But four epistatic QTLs with increasing effects were from Pokkali.

#### QTLs for SIS

A total of 11 chromosomal regions with significant additive effect were detected on chromosomes 2, 5, 6, and 11 by IM. All QTLs are having small effects of at least 5 % but not more than 9 % of the phenotypic variation. Three QTLs were mapped on chromosome 2 (*qSIS2.8, qSIS2.29,* and *qSIS2.28*) with increasing effects from Pokkali alleles. In contrast, ICIM detected seven QTLs. The additive QTLs were distributed on chromosomes 5, 6, 7, 8, 9, and 11. The *qSIS5.1b* was a major QTL, explaining about 13 % of the phenotypic variation. However, *qSIS5.1b* had increasing salt sensitivity effect from Bengal allele. Except for QTLs on chromosome 2, all other additive QTLs had increasing effects due to Bengal alleles. Between the two mapping methods, all QTLs were different except for *qSIS11.2*. For epistatic QTLs, five pairs of interacting QTLs were significant of which four pairs explained 11–15 % of the SIS variation. Among the additive QTLs, *qSIS6.2* was significantly interacting with *qSIS6.30* and increased the PVE from 5 to 15 % (Table 5). All interacting QTLs had increasing effects from Bengal alleles except the *qSIS2.20*.

#### QTLs for Chlorophyll Content

A total of five chromosome regions with additive effects were detected for chlorophyll content under salt stress. Two QTLs were detected on chromosome 11 by IM while ICIM detected two QTLs on chromosome 2 and one QTL on chromosome 3. All additive QTLs were minor-effect QTLs, with increasing CHL effects from Pokkali alleles except *qCHL2.20*. In contrast, epistatic QTL mapping detected ten significant pairs of interacting QTLs. Eight QTL pairs had large effects with PVE as high as 36 %. All additive QTLs were independent of epistatic QTLs for CHL.

#### QTLs for Shoot Length

Six additive QTLs were detected by IM and another six QTLs were detected by ICIM. The *qSHL1.38* and *qSHL3.34* were significant QTLs in both methods. The *qSHL1.38* was a major QTL with LOD value of 37 and accounted for 48–52 % of the phenotypic variation. The additive effect of *qSHL1.38* had increasing effect from Pokkali allele. Other SHL QTLs were located on chromosome 2, 3, 5, and 12 with small effects. Seven pairs of QTLs were significant in epistatic QTL mapping. Five pairs had 11 % PVE and the other two pairs had 9 % PVE. There was no epistatic QTL that co-localized with additive QTL.

#### QTLs for Root Length

Twelve additive QTLs were detected for root length by IM. In contrast, ICIM detected only three QTLs, with *qRTL1.26* common in both methods. Two large-effect QTLs in chromosome 3 (*qRTL3.7* and *qRTL3.10*) were highly significant and accounted for 10 and 12 % of the phenotypic variation, respectively. Both QTLs had increasing effects from Bengal alleles. All other QTLs were minor-effect QTLs, with increasing effects contributed by Bengal allele. Five significant pairs of interacting QTLs with PVE ranging between 9 and 17 % were detected. None of the interacting QTLs were found similar or co-localizing to additive QTLs.

#### QTLs for dry Weight

For shoot dry weight, nine additive QTLs were significant by IM. Three QTLs located on chromosome 5 *(qDWT5.2, qDWT5.4* and *qDWT5.5*) were large-effect QTLs that accounted for 11, 15 and 15 % of the phenotypic variation, respectively. Other QTLs were distributed on chromosomes 1, 4, 6, and 11, with PVE of at least 5 %. In contrast, ICIM detected five significant QTLs for DWT. Two QTLs (*qDWT4.32* and *qDWT5.4*) were common in both methods. Among the five QTLs by ICIM, *qDWT5.4* had the largest effect (PVE = 13 %) with LOD score of 7.6. All DWT additive QTLs had increasing effects coming from Pokkali alleles. Analysis of epistatic QTLs detected six pairs of interacting QTLs. All pairs of interacting QTLs except *qDWT4.16* and *qDWT10.19* had large effects of at least 10 % PVE. Intervals of all epistatic QTLs were independent of additive QTLs.

#### QTLs for Shoot-to-Root Ratio

Additive QTL mapping by IM detected three large-effect and two small-effect QTLs located on chromosomes 1 and 2. The *qSRR1.382, qSRR1.36* and *qSRR2.28* were highly significant and had PVE of 23, 13 and 11 %, respectively. Conversely, ICIM method identified five significant additive QTLs. Among the QTLs, two were large-effect QTLs (*qSRR1.386* and *qSRR2.33*) with PVE of 22 and 11 %, respectively. Pokkali alleles had increasing effects in all additive QTLs for SRR. For interacting QTLs, five large-effect QTL pairs of Bengal and Pokkali origin were detected. All interacting QTLs were mapped to chromosomal regions different from additive QTLs.

#### Quality and Accuracy of QTL Mapping

Segregation distortion is commonly observed in populations developed from crosses between *indica* and *japonica* rice varieties. We mapped the regions of segregation distortion to determine if significant SDLs co-localized to the QTLs detected in this study. Interval mapping for SDLs detected 16 significant intervals that were skewed toward either parent (Table 6). For each chromosome, at least one SDL was mapped, except on chromosomes 2, 4, and 12. In most of the SDLs, Pokkali allele transmission was favored. In chromosome 11 alone, four significant intervals showed segregation distortion favoring inheritance of Pokkali alleles. The average interval size of SDLs was about 198Kb, with the smallest and largest interval size of 600 bp (*sdl11.26*) and 1.4 Mb (*sdl9.12*), respectively. By comparing the positions of QTLs against the positions of SDLs, the additive QTL *qK1.8* and epistatic QTL *qCHL9.12* overlapped exactly with *sdl1.8* and *sdl9.12* intervals. Therefore, these two QTLs should be considered with caution as they deviate from the expected 1:1 segregation ratio in the RIL population. The Bengal allele was transmitted to progeny lines more frequently than the Pokkali allele in *sdl1.8*. In contrast, Pokkali allele was favorably inherited in *sdl9.12*. Overall, most additive and epistatic QTLs mapped in this study were in chromosomal regions not affected by segregation distortion.

**Table 6:** Interval mapping of segregation distortion loci (SDLs) in Bengal/Pokkali F_6_ RIL population

SDL	Chromosome	Position (cM)	Left Marker	Right Marker	Interval	LOD	Segregation ratio	
					size (bp)		Bengal	Pokkali
*sdl1.8*	1	63	S1_8656025	S1_8901503	245,478	7.0103	1	0.419
*sdl1.12*	1	74	S1_12394007	S1_12414777	20,770	6.6211	1	0.4304
*sdl3.29*	3	153	S3_29855008	S3_30045852	190,844	4.0197	0.5244	1
*sdl3.34*	3	181	S3_34487907	S3_34521908	34,001	3.4639	0.5504	1
*sdl5.22*	5	96	S5_22077219	S5_22142421	65,202	3.2006	0.5639	1
*sdl6.4*	6	23	S6_4269744	S6_4327404	57,660	3.2649	0.5605	1
*sdl6.9*	6	57	S6_9246940	S6_9317830	70,890	3.0751	1	0.5706
*sdl7.26*	7	109	S7_26680214	S7_26796826	116,612	2.5927	0.5983	1
*sdl8.7*	8	43	S8_7488739	S8_7668333	179,594	29.5389	0.1136	1
*sdl8.16*	8	52	S8_16619372	S8_16941109	321,737	22.0004	0.1761	1
*sdl9.12*	9	29	S9_12915373	S9_14359383	1,444,010	13.3385	0.2847	1
*sdl10.12*	10	31	S10_12765359	S10_12968073	202,714	2.5777	0.5992	1
*sdl11.17*	11	61	S11_17286328	S11_17316420	30,092	3.5648	0.5455	1
*sdl11.22*	11	91	S11_22242895	S11_22274274	31,379	2.8801	0.5814	1
*sdl11.23*	11	101	S11_23708208	S11_23866022	157,814	2.9439	0.5778	1
*sdl11.26*	11	115	S11_26254930	S11_26255530	600	5.3304	0.4724	1

Plant height is one of most frequently studied traits in QTL mapping. Several studies showed that plant height has high heritability and stable at different growth stages at different environments (Yan et al. 1998). In rice, 1011 QTLs were reported for plant height (gramene.org). Among these QTLs, *sd1* is the main QTL that played a major role in the development of semi-dwarf varieties in rice (Khush 1999). To assess the quality of our phenotypic data and the accuracy of our QTL mapping, we surveyed plant height QTLs in rice under normal or stress conditions and compared the positions of our SHL QTLs to see if we can detect any of the previously reported plant height QTLs. In both mapping methods, the green revolution gene *sd1* gene, LOC_Os01g66100 (Spielmeyer et al. 2002) was located within our major QTL designated as *qSHL1.38*, with LOD value as high as 36 and PVE of 51 %. The *sd1* gene is about 95 Kb away from the left SNP marker and 226 Kb from the right SNP marker of *qSHL1.38*. Moreover, *qSHL12.25* was found within the region of *qPHT12-1* on chromosome 12 located between 23,603,156-26,017,884 bp region (Hemamalini et al. 2000). Also, *qSHL3.34* was covered within the interval of *QPh3c* located between 32,945,649-36,396,286 bp of chromosome 3 (Li et al. 2003). The minor QTL *qSHL1.7* was flanked within *ph1.2* located in 5,941,464-7,445,919 bp region on chromosome 1 (Marri et al. 2005); while *qSHL2.18* was found within the reported QTL on chromosome 2 at 17,484,665-33,939,159 bp region (Huang et al. 1996). Additionally, *qSHL5.6* was confirmed within the QTL region of chromosome 5 located in between 5255, 880-6,700,408 bp region (Mei et al. 2003) and in *ph5* located between 6,132,767-18,875,558 bp region on chromosome 5 (Zhuang et al. 1997). In summary, the locations of six *SHL* QTLs matched with previously reported plant height QTLs. In addition, four new minor QTLs were mapped in this study, each contributing at least 5 % of the plant height variation. Together with other QTLs for other traits, a total of eleven QTLs in this study were validated (Table 7). Therefore, our QTL mapping by IM and ICIM methods using ultra-high density genetic map is robust and informative.

**Table 7:** Summary of additive QTLs co-localizing to previously reported QTLs

Trait	QTL in this study	Previous QTL	Reference
K^+^ concentration	*qK1.11*	*qSKC1*	Thomson et al. (2010)
	*qK6.4*	*QTL on chr. 6, at 30 cM*	Koyama et al. (2001)
NaK ratio	*qNaK1.11*	*qSNK1*	Thomson et al. (2010)
		*QTL on chr. 1, at 74 cM*	Koyama et al. (2001)
Salt injury score	*qSIS9.8*	*qSES9*	Thomson et al. (2010)
Plant height	*qSHL1.38*	*sd1*	Spielmeyer et al. (2002)
	*qSHL1.7*	*ph1.2*	Marri et al. (2005)
		*qPH1.2*	Bimpong et al. (2013)
	*qSHL2.18*	*QTL on chr. 2 at 17-33 Mb*	Huang et al. (1996)
	*qSHL3.34*	*QPh3c*	Li et al. (2003)
	*qSHL5.6*	*ph5*	Zhuang et al. (1997)
		*QTL on chr. 5 at 5.2- 6.7 Mb*	Mei et al. (2003)
	*qSHL12.25*	*qPHT12-1*	Hemamalini et al. (2000)
Shoot dry weight	*qDWT6.24*	*qDWT6.1*	Bimpong et al. (2013)

#### Identification of Candidate Genes Within QTLs

**Fig. 3:**
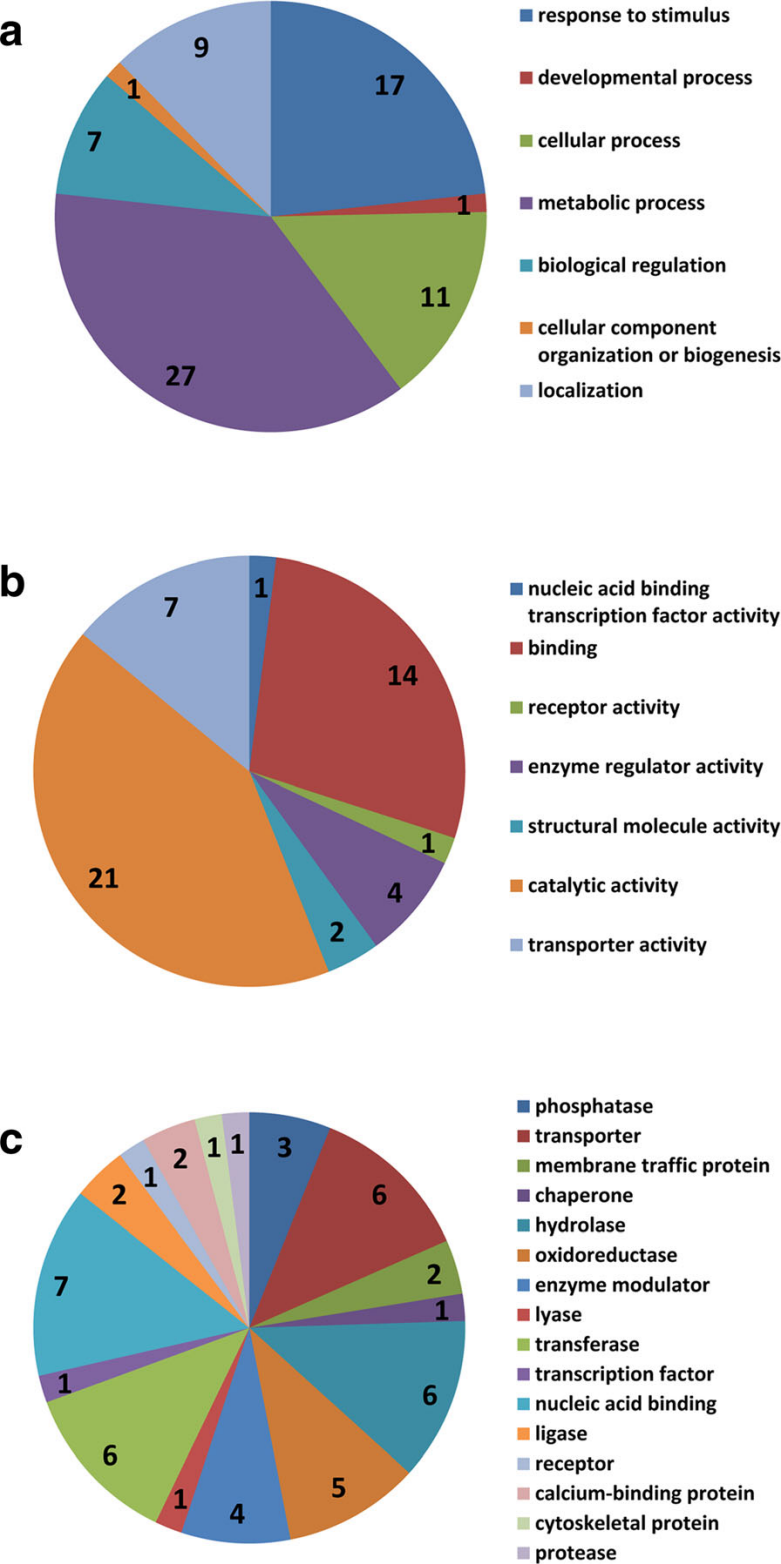
Functional classification of annotated candidate genes delimited by additive QTLs for salinity tolerance. **a** classification by biological class; **b** classification by molecular function; **c** classification by protein class

The saturation of SNP markers in our linkage map allowed us to detect QTLs at an interval size much shorter than previously reported QTLs. In this study, the average interval size of a QTL was 132 Kb, with minimum and maximum interval size of 22 bp and 877 Kb, respectively (Table 4). For nine traits, IM and ICIM mapped 64 and 36 additive QTLs. Fifteen QTLs were commonly detected in both methods with a total of 85 QTLs. To identify candidate genes underlying fitness of rice under salt stress, we looked at all genes in the QTL region using flanking markers. For 36 additive QTLs by ICIM, a total of 704 genes were present within QTLs (Additional file 2: Table S3), of which, 110 were annotated while the 594 genes were identified as expressed proteins, hypothetical proteins, transposon, and retrotransposon proteins. Similarly, for 64 additive QTLs identified by IM method, only 111 of 1046 genes were annotated. For the 1344 gene models in the 85 QTLs for nine traits, 79 genes were classified in 7 biological processes, 50 genes were classified into 7 molecular functions, and 49 genes were classified into 16 protein classes (Fig. 3). A large portion of the candidate genes was involved in metabolic processes and responses to stimuli. Candidate genes classified in biological regulation and localization (six transporters) were found within QTLs.

## Discussion

QTL mapping has been implemented in many breeding programs to discover genes underlying quantitative traits. However, many of these reported QTLs covered large chromosome intervals, thus, limiting the application of flanking markers in predicting the phenotype of the plant. A major constraint to previous QTL mapping studies is the number of available polymorphic markers. However, with reduction in DNA sequencing cost, high resolution QTL mapping is now possible using SNP markers. In this study, we utilized the GBS approach to develop an ultra-high-density genetic linkage map of rice for identification of QTLs for traits related to salinity tolerance. Thirty-eight SNP calls segregating in the RIL population were validated by re-sequencing the target region in both parents. Out of 38 SNP markers, only one SNP call in Bengal was not in agreement (Additional file 3: Table S2). Therefore, the GBS data have high quality SNP calls for linkage and QTL mapping. In spite of the large number of SNP markers placed on the linkage map, there were 20 gaps of about 5 cM intervals. These gaps could be due to removal of SNP markers during filtering process. Due to multiplexing of large number of DNA samples in the GBS, representation of a SNP in all samples was greatly reduced resulting in the removal of more than two-thirds of the GBS data. The linkage map closely resembled the rice genetic map of Harushima et al. (1998). Mapping of segregation distortion loci using this map indicated 16 intervals showing segregation distortion (Table 6). Two SDLs co-localized to QTLs for salinity tolerance (*qK1.8* and *qCHL9.12*). Therefore, genetic variances contributed by these QTLs may not be accurate due to segregation distortion. In addition to availability of numerous SNP markers for linkage map construction, the quality of phenotypic estimates is equally important for QTL mapping. We assessed this by comparing our shoot length QTLs with reported plant height QTLs. Ten QTLs for SHL were detected (Table 4), of which, six QTLs for plant height including the major *sd1* (*qSHL1.38*) co-localized to previously reported plant height QTLs. Validation of those QTLs suggests that our phenotypic and genotypic data for QTL mapping are of high quality (Table 7). With five to six markers per cM, the average QTL interval size was 132Kb. The maximum resolution of QTL was about 22 bp interval (*qSIS6.20*) and the largest QTL interval size was about 877Kb (*qSIS11.2)* (Table 4).

Previous QTL mapping studies for salinity tolerance mainly focused on detecting additive QTLs despite the complex nature of salinity tolerance. In this study, we also mapped interacting QTLs significantly contributing to the phenotypic variation of each trait under salt stress (Table 5). Di-genic interval mapping for epistatic QTLs revealed interaction of alleles from Pokkali and Bengal. In general, interacting QTLs were located in chromosome intervals independent of additive QTLs. Likewise, the variance explained by epistatic QTL pair was higher than the variance explained by individual additive QTL. For example, additive QTLs for Na^+^ concentration and CHL revealed very few small-effect QTLs. In contrast, many of the epistatic QTL pairs for Na^+^ and CHLs had larger PVE as high as 35 %. Therefore, these findings indicated the importance of epistatic QTLs in salt stress response in rice. Many of the QTLs flanked small intervals with few candidate genes. Overall, the ultra-high density genetic map and the high-quality phenotypic data facilitated a high resolution QTL mapping for salinity tolerance. In addition, the genetic map will be useful in discovery of novel QTLs for other contrasting agronomic traits between Bengal and Pokkali.

Since the beginning of the search for QTLs underlying salinity tolerance, Na^+^ concentration, K^+^ concentration, NaK ratio, and salt injury score were often investigated. Similar to previous reports, Na^+^ concentration was highly correlated to SIS or standard evaluation score (SES) and survival of rice plants under salt stress (Yeo et al. 1990; Platten et al. 2013). The shoot Na^+^ concentration also had significant positive correlation to NaK ratio and shoot K^+^ concentration (Table 2). The Na^+^ and K^+^ relationship implies that as shoot Na^+^ concentration increases, shoot K^+^ concentration also increases. It is likely that during salt stress, many lines do not discriminate these cations, thus, suggesting possible accumulation of Na^+^ and K^+^ in the shoot through non-selective cation channels (Demidchik and Maathuis 2007). This is evident in the high heritability of Na^+^ and K^+^ concentrations in the population (Table 1). In previous studies of QTLs for shoot Na^+^ concentration, QTLs were mapped on chromosomes 1 (Thomson et al. 2010), 3, 9, 11, (Wang et al. 2012), 4 (Koyama et al. 2001), and 7 (Lin et al. 2004). None of our additive QTLs for Na^+^ concentration co-localized to previous QTLs, but, the epistatic QTL *qNa6.4* is possibly the same additive QTL in chromosome 6 at 24 cM (Koyama et al. 2001). The effects of additive QTLs for Na^+^ concentration were all minor. Surprisingly, four pairs of interacting intervals had significant larger effects (11–15 % PVE), suggesting that interactions among Na^+^ QTLs were important in the accumulation of Na^+^ in shoot. Alleles of Na^+^ QTLs from both parents contributed to shoot Na^+^ accumulation. In contrast, all alleles of additive QTLs for shoot K^+^ concentration were from Pokkali (Table 4). Therefore, it is interesting to know the underlying genes for K^+^ accumulation and their role in accumulation of other cations like Na^+^. The presence of transgressive segregants exhibiting higher concentration of shoot K^+^ and lower NaK ratio than Pokkali suggests the presence of positive alleles in both parents for selective cation transport during salt stress (Fig. 1). In case of Pokkali, salt tolerance response could be due to maintenance of high K^+^ concentration or low NaK ratio (Ren et al. 2005) and by compartmentalization of Na^+^ ions into the shoot vacuoles (Kader and Lindberg 2005). The strong relationship among Na^+^, K^+^, and SIS prompted us to look for the co-location of QTLs underlying these traits. Our result showed that *qNa6.5* and *qNaK6.5*, *qK1.11* and *qNaK1.11*, and *qSIS6.2* and *qNaK6.2* co-localized in the same intervals (Table 4). Therefore, it is possible that these traits shared the same underlying causal genes. The co-location of *qNa6.5* and *qNaK6.5* is more likely not coincidental because both alleles of the two QTLs came from Bengal and had increasing effect in the concentration of Na^+^ ions. On the other hand, the co-location *of qK1.11* and *qNaK1.11* is consistent with co-location of shoot K^+^ concentration, *SKC1* and shoot Na^+^/K^+^ ratio, *SNK1* (Thomson et al. 2010, Wang et al. 2012). Allele substitution of Bengal with Pokkali at *qK1.11* had increasing effect in the shoot K^+^ concentration. In contrast, Bengal allele of *qNaK1.11* had increasing effect on NaK ratio, thus, corroborating the desirability of Pokkali allele at the locus for salt tolerance. In previous studies, *SKC1* was responsible for 10–40 % of the variation in shoot K^+^ concentration (Koyama et al. 2001; Lin et al. 2004; Thomson et al. 2010; Wang et al. 2012). Here, the *qK1.11* accounts for only 16 % of the variation. The discrepancy in the estimation of PVE is likely attributed to differences in population size and number of markers used in different studies. The q*K1.11* is covering a 52Kb interval between 11.52–11.58 Mb region on chromosome 1 with six genes. This interval is within the reported *SKC1* by Thomson et al. (2010), but, downstream of 11.46 Mb region of the cloned *HKT1;5* (Ren et al. 2005). While Thomson et al. (2010) assumed *HKT1;5* (LOC_Os01g20160) as the underlying gene for *qSKC1* or *Saltol,* it is also possible that other genes contributing toward salt tolerance might be present in the *SKC1* region. This possibility is supported by the findings from a genome-wide association mapping study (Kumar et al. 2015), where 12 significant SNPs were located between 9.6 and 14.5 Mb region of chromosome 1. One of the 12 SNPs with high linkage disequilibrium (LD) at 11.6 Mb region (1:11608731) is 26Kb away from the right marker of *qK1.11*. Furthermore, *HKT1;5* allele mining in several rice cultivars showed a weak association of *HKT1;5* allele to low Na^+^ concentration to account for salinity tolerance. The *HKT1;5* allele in aromatic group that included Pokkali showed low Na^+^ concentration. However, several cultivars having different *HKT1;5* alleles (Aus, FL478, Hasawi, Daw, Japonica lines, and *O. glaberrima)* also showed low Na^+^ concentration and high salt tolerance (Platten et al. 2013). Additionally, our genetic map data showed the availability of markers that flanked *HKT1;5* gene (Additional file 1: Table S1, at 70.2 cM) and the absence of segregation distortions in these regions (Table 6), but the IM and ICIM methods both detected QTL for high shoot K^+^ concentration downstream of *HKT1;5*. Interestingly, the *qK1.11* interval contained two transposons, three uncharacterized expressed proteins, and a CC-NBS-LRR-encoding gene (LOC_Os01g20720). NBS-LRR genes are the largest class of resistance genes implicated in the recognition of pathogen-derived avirulence protein. In rice, a gene encoding a CC-NBS-LRR, *Pb1,* provided a durable panicle blast resistance by interacting with WRKY45 transcription factor for the activation of signal transduction pathway (Inoue et al. 2013). On the other hand, overexpression of *ADR1* gene encoding a CC-NBS-LRR in *A. thaliana* showed enhanced drought tolerance (Chini et al. 2004). Therefore, the role of LOC_Os01g20720 gene in signal transduction pathway and shoot K^+^ ion accumulation should be investigated. Other QTLs for shoot K^+^ concentration such as *qK1.8, qK1.38, qK5.4, qK6.4,* and *qK6.5* covered at least 250 kb intervals containing 33, 40, 86, 61, and 34 gene models, respectively. Candidate genes present in these QTL intervals include protein kinases, transcription factors, ethylene, auxin-responsive proteins, flavin-containing monooxygenases, and several expressed proteins of unknown function. In contrast, *qNa2.7* is saturated with transposons and retrotransposons except for a putative membrane lipid channel, scramblase protein (LOC_Os02g14290). The *qNa12.18* flanked four transposons and a hypothetical protein.

For NaK ratio QTLs, the co-location of *qSIS6.2* and *qNaK6.2* confirmed the significant correlation of NaK ratio to SIS. For both QTLs, Bengal alleles were undesirable. Only seven genes including a WRKY113 transcription factor (LOC_Os06g06360) were present in this QTL interval. Whether WRKY113 is interacting with the CC-NBS-LRR in *qK1.11* or *qNaK1.11* like the *Pb1*, presents an interesting perspective to study gene interactions and salt tolerance. In contrast, the large-effect *qNaK6.5* (or *qNa6.5*) still covered a 264Kb interval and contained 34 gene models. Candidate genes in this interval are MYB transcription factor (LOC_Os06g10350), cyclic nucleotide-gated ion channel (LOC_Os06g10580), transcription elongation factor SPT5 (LOC_Os06g10620), and leaf senescence-related protein (LOC_Os06g10560). Among the NaK QTLs, *qNaK1.11* is likely the same QTL as *qSNK1* (Koyama et al. 2001; Thomson et al. 2010).

SIS reflects the overall plant’s response to salt stress. Hence, we are particularly curious in finding QTLs to identify underlying genes for this trait. Among the additive QTLs, *qSIS5.1b* had PVE of 13 % with increasing effect from Bengal allele. Therefore, in breeding for low SIS, the corresponding Pokkali allele at *qSIS5.1b* is desirable. The variance explained by *qSIS6.2* alone was only 5 %, but, interaction to *qSIS6.30* increased the PVE to 15 % (Table 5). This result indicated the additive and epistatic effect of a locus and emphasized the importance of QTL interactions in understanding the complexity of SIS or salt tolerance. Among previously mapped QTLs for salt evaluation score (SES) or salt tolerance rating (STR), the *qSIS9.8* is located within the interval of *qSES9* (Thomson et al. 2010). The *qSIS2.8* interval contained 25 genes, one of which encoded a cyclic nucleotide-gated ion channel (LOC_Os02g15580). In contrast, *qSIS5.1b* and *qSIS6.20* contained two and one gene, respectively. Both QTLs delimited a lectin protein kinase (LOC_Os06g35870, LOC_Os05g03450). In *A. thaliana*, lectin protein kinases were involved in the protein-protein interactions for structural stability of plasma membrane and plant cell wall (Gouget et al. 2006). Therefore, it will be interesting to see if plasma membrane stability conferred by lectin protein kinase enhances salinity tolerance. Similarly, the *qSIS6.21* interval confined a single candidate gene that encodes a receptor-like protein kinase 5 precursor (LOC_Os06g36270). In *qSIS5.03*, a vacuolar ATP synthase (LOC_Os05g01560) is one of the four genes in the interval while a trehalose phosphatase is one of the five candidate genes in *qSIS5.1a*. In rice, transcript expression of a mitochondrial ATP synthase (RMtATP6) was induced in leaves by NaCl and NaHCO_3_ treatments and overexpression of RMtATP6 in tobacco plants showed enhanced seedling salt tolerance (Zhang et al. 2006). On the other hand, overexpression of trehalose-6-phosphate phosphatase and trehalose-6-phosphate synthase increased tolerance to drought, salt, and cold in rice (Jang et al. 2003). Also, of great interest is the *qSIS6.7* interval that delimited only three genes including a pyrophosphate fructose-6-phosphate 1-phosphotransferase (LOC_Os06g13810) and a flavin monooxygenase in *qSIS7.14*. Pyrophosphate: fructose-6-phosphate 1-phosphotransferase was associated to seedling salt tolerance (Lim et al. 2014) while overexpression of a flavin monooxygenase designated as YUCCA enhanced drought tolerance of *A. thaliana* (Cha et al. 2015). Additionally, *qSIS8.24*, *qSIS9.8,* and *qSIS11.2* delimited genes involved in signal transduction pathway.

Plant vigor under salt stress is a good predictor of tolerance. In addition to common traits investigated under salt stress, CHL, and growth parameters such SHL, RTL, SRR, and DWT were also examined. In soybean, salinity tolerance was determined by a major QTL for chlorophyll content (Patil et al. 2016). In contrast, additive QTLs for CHL were all minor-effect QTLs while several pairs of epistatic QTLs had PVE as high as 35 % (Tables 4 and 5). Comparison of CHL QTLs with earlier reported QTLs co-localized *qCHL2.20* and *qCHL3.26* within the intervals of *qCHL2* and *qCHL3* (Thomson et al. 2010). All other CHL QTLs are novel, thus, offering new targets for further analysis. The *qCHL3.26* interval flanked a single unknown expressed protein (LOC_Os03g47190) while *qCHL2.20* contained six retrotransposons and one expressed protein. Aldehyde dehydrogenase (LOC_Os02g49720) and zinc-knuckle family protein (LOC_Os02g49670) were found in *qCHL2.30* interval. *Arabidopsis* plants overexpressing aldehyde dehydrogenase improved salinity tolerance of plants by reducing the accumulation of reactive oxygen species (Sunkar et al. 2003). Among the 44 genes in the *qCHL11.1,* a NAC transcription factor and a glutathione S transferase are promising candidate genes. In rice, overexpression of a NAC transcription factor showed increased tolerance to drought and salt stress (Zheng et al. 2009). Conversely, glutathione S-transferase had negative effect to drought and salt tolerance in *Arabidopsis* plants (Chen et al. 2012)*.* On the other hand, *qCHL11.2* interval contained seven genes, one of which encodes an HVA22. In barley and *Arabidopsis*, aleurone cells transformed with HVA22 inhibited the formation of GA-induced formation of vacuoles and programmed cell death (Gou and Ho 2008). Since vacuoles are important storage of Na^+^ for salt tolerance, HVA22 is a promising candidate gene for salt tolerance.

Among the SHL QTLs, *qSHL1.38* and *qSHL2.18* were validating the *qPH1.2* (Bimpong et al. 2013) and *qPH2* (Thomson et al. 2010), respectively, for plant height QTLs investigated under salt stress. The SHL QTLs contained many candidate genes. In addition to the major *sd1* gene within *qSHL1.38*, other candidate genes were AP2 domain containing protein (LOC_Os01g04020) in *qSHL1.1*, KH domain containing protein (LOC_Os01g13100) in *qSHL1.7a,* auxin response factor1 in *qSHL1.7b,* potassium transporter (LOC_Os01g13520) in *qSHL2.18*, gibberellin 2-oxidase (LOC_Os05g06670) in *qSHL5.3,* gibberellin 3-beta-dioxygenase (LOC_Os05g08540), cytokinin-O-glucosyltransferase (LOC_Os05g08480) and auxin OsIAA15 (LOC_Os05g08570) in *qSHL5.4,* OsMAD66 transcription factor (LOC_Os05g11380) in *qSHL5.6,* and OsSAUR57 in *qSHL12.25*. A putative RNA-binding protein containing a KH domain was reported to be important in *Arabidopsis* plants for heat stress tolerance (Guan et al. 2013). In other plants, AP2/ERF transcription factors were implicated in the control of metabolism, growth, and development, and in responses to environmental stress (Licausi et al. 2013).

The relationship of Na^+^ concentration with SHL, RTL, DWT, and SRR were not significant. However, correlation of these traits to SIS, indicated growth inhibition with increasing sensitivity to salt stress (Table 2). For RTL, large-effect additive QTLs were detected on chromosome 3 (*qRTL3.7, qRTL3.10*) while the rest were minor-effect QTLs located on chromosomes 1, 2, 3, 4, 8, and 9. The majority of root length variation was explained in the epistatic QTLs. Similarly, QTLs for DWT detected only three large-effect QTLs on chromosome 5 (*qDWT5.2, qDWT5.4, qDWT5.5*) and all epistatic QTL pairs had PVE not lower than 10 %. In contrast, five large-effect additive QTLs were mapped on chromosomes 1 and 2 for SRR. The *qSRR1.382* was located on the same interval of *qSHL1.38* and so, the same *sd1* gene determined the increased SRR. The fact that all DWT and SRR additive QTLs were contributed by Pokkali suggested the growth-increasing effect of Pokkali alleles under salt stress. On the other hand, the significant epistatic QTLs identified in all traits emphasized the importance of additive and epistatic effects for salinity tolerance.

The growth of roots during seedling stage under salt stress was not investigated before. All RTL QTLs in this study were new QTLs. A total of 117 genes models was delimited by 14 QTLs. In *qRTL1.22,* only two gene models were present, a retrotransposon and an uncharacterized expressed protein. Of particular interest is the VQ domain containing protein (LOC_Os01g46440) within *qRTL1.26*. In *Arabidopsis*, VQ-containing proteins interact with WRKY transcription factors and negatively regulate plant resistance to pathogen infection (Wang et al. 2015). Other candidate genes within RTL QTLs are aldehyde dehydrogenase (LOC_Os02g43194) and polyamine oxidase (LOC_Os02g43220) in *qRTL2.26,* ankyrin repeat-reach protein (LOC_Os02g54860) and trehalose-6-phosphate (LOC_Os02g54820) among seven genes contained in *qRTL2.33,* an integral membrane protein (LOC_Os03g11590) in *qRTL3.6,* MYB transcription factor (LOC_Os03g13310) and transporters (LOC_Os03g13240, LOC_Os03g13250, LOC_Os03g17740) in *qRTL3.7* and *qRTL3.* An asparagine synthetase (LOC_Os03g18130) is within *qRTL3.10*, while a vacuolar protein sorting-associated protein 18 (LOC_Os08g08060), transporter (LOC_Os08g08070), and an RLK gene (LOC_Os08g08140) are delimited in *qRTL8.4*. The *qRTL9.14* contains only three genes, one of which is a WRKY gene (LOC_Os09g25060). The *qRTL8.27* contains a PDR ABC transporter gene (LOC_Os08g43120).

Koyama et al. (2001) detected one QTL for dry mass on chromosome 6 at 34 cM. A total of six DWT QTLs were mapped on chromosome 6 by IM and ICIM. However, none of our QTLs are localized at 34 cM region. The *qDWT6.24*, however, validated the *qDWT6.1* detected by Bimpong et al. (2013). Notable candidate genes within DWT QTLs are transporters (LOC_Os01g38670, LOC_Os01g38680, LOC_Os05g04600, LOC_Os05g08430) in the intervals of *qDWT1.2, qDWT5.2,* and *qDWT5.4*, calmodulin-binding transcription factors (LOC_Os01g69910, LOC_Os05g10840) in *qDWT1.40*, a REX1 DNA repair gene (LOC_Os05g10980) in *qDWT5.5*, a MYB transcription factor (LOC_Os06g02250) in *qDWT6.06*, and a lectin protein kinase (LOC_Os06g35870) in *qDWT6.20*. In addition, a calcium-binding mitochondrial carrier (LOC_Os06g40200) is within *qDWT6.23* while an ABC-type transporter gene (LOC_Os06g40550) is in *qDWT6.24*.

SRR QTLs under salt stress were not investigated in previous QTL mapping studies. All QTLs for SRR are new QTLs for further understanding of plant’s fitness under salinity stress. The large effect QTL *qSRR1.36* spanned five genes including a WRKY119 gene (LOC_Os01g62510). The *qSRR1.382* and *qSRR1.386* contained an amino acid transporter (LOC_Os01g66010) and several receptor-like protein kinases. In contrast, *qSRR2.31* delimited a single expressed protein. Again, a trehalose-6-phosphate (LOC_Os02g54820) and ankyrin repeat rich protein (LOC_Os02g54860) are two of the seven genes found in the *qSRR2.33* interval while a HEAT repeat protein is within *qSRR2.34* interval and another transporter is located in *qSRR3.9*. In addition to few candidate genes with known functions present within small-effect QTLs *qSRR3.10, qSRR3.11, qSRR4.10, qSRR8.19,* and *qSRR8.26*, there were several uncharacterized expressed proteins.

Taken together, at least six transporter genes were located within six QTLs, of which, three transporter genes were found in QTLs for root length (LOC_Os3g11590 in *qRTL3.6*; LOC_Os3g17770 in *qRTL3.9*, and LOC_Os3g11590 in *qRTL3.7*), while one transporter gene was contained in *qSIS11.2* (LOC_Os11g06810), *qCHL11.2* (LOC_Os11g05800), and *qSHL3.34* (LOC_Os03g61290). In addition to transporters and genes for detoxification or osmotic adjustment (flavin monooxygenase, trahalose-6-phosphate), the prevalence of protein kinases suggest the role of signal transduction pathway and possible regulation of biological and cellular processes by transcription factors (Fig. 3).

## Conclusion

The availability of ultra-high density genetic map and robust phenotypic data enabled us to identify additive QTLs with high resolution and facilitated identification of candidate genes. Detection of significant epistatic QTLs in addition to additive QTLs validated the complex architecture of salinity tolerance, which is possibly determined by concerted interactions of several genes. While *Saltol* or *SKC1* may provide salinity tolerance and already being introgressed to several rice varieties in Asia, it may not provide adequate tolerance to salt stress. Our result suggested the use of multiple QTLs, especially the genes for low salt injury score to enhance salinity tolerance. The candidate genes identified in this study will be useful targets for functional genomics, gene-pyramiding, and gene-based marker-assisted breeding. Our study demonstrated the power and application of GBS for QTL mapping of a complex genetic trait like salinity tolerance.

## Methods

### Plant Materials and Population Development

A mapping population was developed by crossing Bengal and Pokkali as female and male parent, respectively. The resulting F_1_ lines were selfed and advanced by single seed descent method to generate 230 recombinant inbred lines (RILs) in F_6_ generation. RILs grown in unsalinized condition were extracted for DNA and were genotyped by the Cornell Genomic Diversity Facility using the GBS method.

### Phenotypic Characterization and Tissue Collection

The phenotypic evaluation was conducted in the greenhouse with day time and night time temperature settings at 26–29 °C. The hydroponics system was used in the screening for seedling salt tolerance following the IRRI standard evaluation technique (Gregorio et al. 1997). The parental lines and 230 RILs were pre-germinated in a paper towel for 2 days and then transplanted to hydroponic set up containing 1 g/L of Jack’s Professional (20-20-20) (J.R. Peters, Inc.), supplemented with 300 mg/L of ferrous sulfate. The pH of the solution was maintained at 5.0–5.1 and plants were allowed to grow for 2 weeks. The whole experiment was conducted in randomized complete block design replicated three times, with ten plants per line per replicate.

At 14^th^ day after planting, the plants were subjected to 6dSm^-1^ for 2 days and then into 12dSm^-1^ salt stress. After 6 days of salt stress, the amount of chlorophyll content was measured on the mid-length of the second youngest leaf using a SPAD-502 chlorophyll meter (Spectrum Technologies, Inc.). Five plants per line of uniform growth were evaluated for traits related to salinity tolerance. On the 9^th^ or 11^th^ day, when the susceptible check plants were dead, lines were phenotyped for salt injury score, shoot length, and root length. A score of 1 was given to unaffected plants, score of 3 to healthy plants but stunted, score of 5 to plants showing green leaves and stem with some tip burning and leaf rolling, score of 7 to plants with green stem but all leaves are dead, and a score of 9 to completely dead plants. Shoot length and root length were measured in centimeter. Shoot length was measured from the base of the culm to the tip of the tallest leaf. Root length was measured from the base of the culm to the tip of the longest root. Shoot length to root length ratio was derived by dividing the shoot length by the root length. For dry weight, five plants per line were collected and dried at 65 °C oven for 5 days prior to weighing.

### Measurement of Na^+^ and K^+^ Concentration in Shoot

The amount of Na^+^ and K^+^ in the shoot was measured from 100 mg ground tissue taken from a pool of five plants per line. Briefly, the shoots of the plants were collected, rinsed with water, oven dried for 5 days and ground to fine powder. The tissue was digested with 5 ml of nitric acid and 3 ml of 30 % hydrogen peroxide at 152–155 °C heating block for 3 h (Jones and Case 1990). The digested tissue was diluted to a final volume of 125 ml. Flame photometer (model PFP7, Bibby Scientific Ltd, Staffordshire, UK) was used to quantify the Na^+^ and K^+^ concentrations in each sample. The final concentrations were computed from the derived standard curve of different dilutions of Na^+^ and K^+^ and the ratio of Na^+^ and K^+^ concentration (NaK) was calculated from these values.

### Statistical Analyses

The phenotypic data for each trait were analyzed by ANOVA and LS mean of each line was extracted using the GLIMMIX procedure. The RIL line was entered as a fixed effect and replication as a random effect. Broad sense heritability for each trait was computed by family mean basis following Holland et al. (2003). CORR procedure was implemented to determine the relationship among traits. All data analysis was conducted using Statistical Analysis System (SAS) software version 9.4 for Windows (SAS Institute Inc 2012). Frequency distribution for each trait was constructed in Microsoft Excel 2010.

### Genotyping-by-Sequencing of Bengal, Pokkali, and RIL Population

Leaf tissues were collected from each of the parental lines and RIL. The DNA was extracted using the Qiagen DNeasy Plant Mini Kit following the manufacturer’s protocol (Qiagen Inc., Valencia, CA, USA). Genomic DNA libraries were prepared as described by Elshire et al. (2011). Each DNA was cut by *ApeKI* enzyme and the adapters were ligated to barcode the DNA of each line. Pooled DNA from parents, 189 RILs, 94 other lines and 3 blanks was sequenced in one lane with the Illumina HiSeq sequencer at Genomic Diversity Facility, Cornell University Institute of Biotechnology (http://www.biotech.cornell.edu/brc/genomic-diversity-facility). The Tassel GBS pipeline was used to process the data and SNP calling was based on the Nipponbare reference genome MSU release 7 (Kawahara et al. 2013).

### Construction of Linkage Map and QTL Analysis

Sequence alignment and SNP calling were done by the Genomic Diversity Facility, Cornell University. A total of 1,593,692 tags were sequenced, of which, 1,215,287 (76.3 %) were aligned to unique positions, 134,210 (8.4 %) had multiple alignments and 244,195 (15.3 %) were not aligned. Upon processing and filtering of SNPs, the resulting SNPs markers were reduced to a total of 33,987, with an average individual depth of 5.5 or site depth of 4.6 and individual mean missingness of 0.28. Pokkali and two RILs were declared as failed samples for having less than 10 % of the mean reads per sample. They were removed before further analysis, resulting to a total of 187 RILs for final analysis. The hapmap data file containing the filtered SNP calls were further analyzed prior to linkage map construction and QTL analysis. The Bengal parent was successfully sequenced, thus providing data for differentiation of alleles among RILs. To validate the GBS SNP calling, we amplify and re-sequenced 38 positions of GBS SNP calls in Bengal and Pokkali. Allele differentiation and allele origin among RILs were confirmed with Bengal and Pokkali re-sequenced data available in our laboratory. With the breeding scheme of the mapping population, only three possible genotypes may exist at polymorphic loci with bi-allelic SNP calling. The 2, 0, -1 coding numbers were then used to code for different alleles in the genotype data. SNP call for each marker across the population was coded as 2 if the allele was the same as Bengal. A code of 0 was given to the alternative allele and was assumed as the allele from Pokkali. Since our materials are F_6_ RIL, most of the loci were homozygous and should be segregating into 1:1. However, with low read depth due to highly multiplexed nature of GBS, all heterozygous SNPs (Y = T|C, M = A|C, W = T|A, R = A|G, S = C|G, K = G|T) and missing SNP (N) calls were coded as -1. All SNP markers monomorphic across the 187 RILs were removed. Likewise, all SNP markers with more than 10 % missing SNP calls were purged before further analysis. As a result, only 9303 SNP markers were retained and used for linkage and QTL mapping. The order of SNP markers along the chromosome was fixed based on the physical position of SNPs in the MSU Rice Genome Annotation (Osa1) Release 7. Genetic distances of SNP markers based on recombination rates were converted using the Kosambi mapping function. To see if segregation distortion of markers occurs in the QTLs detected in this study, interval mapping of segregation distortion locus (SDL) was also conducted. Significant SDLs were declared for loci exceeding the 2.0 LOD threshold level.

Nine traits were used for QTL mapping. The mean of three replications was used as phenotypic score for each trait. Except for salt injury score, Na^+^ concentration, K^+^ concentration, Na^+^/K^+^ ratio, chlorophyll content, shoot length, root length, shoot dry weight and shoot length to root length ratio showed normal distribution. Hence, the data were directly used for QTL mapping. For SIS, data were log transformed to improve the normality of RIL distribution prior to QTL mapping. Analysis of additive QTLs for traits related to salinity tolerance was performed by interval mapping (IM-ADD), and inclusive composite interval mapping (ICIM-ADD) methods. By interval mapping method, parameters for QTL detection were set to a scanning window size of every 1 cM with LOD threshold value set at 2.0 to declare significant QTLs. In ICIM-ADD, the parameters were set as follows: missing phenotype by mean replacement, stepwise regression method every 1 cM window size with the probability levels of entering and removing variables set at 0.001, and a second step scanning by interval mapping for significant QTL detection at LOD threshold of 2.0. Epistatic QTLs were identified by interval mapping every 5 cM window with LOD threshold set at 3.0. The phenotypic variation explained by QTLs and their genetic effect were estimated. Confidence interval of each QTL was delimited by the flanking markers within the 1-LOD drop from the estimated QTL position. QTL interval size is computed from the distance between the physical positions of left and right flanking markers. Significant QTL for each trait was named with the trait, followed by numbers indicating the chromosome location and megabase (Mb) position of the QTL. For example, *qK1.8* indicates the presence of a QTL for shoot K^+^ concentration in chromosome 1 located at 8 Mb region. All linkage, SDL and QTL analyses were implemented in QTL IciMapping software version 4.0.6.0 (Meng et al. 2015).

### Candidate Gene Prediction

To identify potential candidate genes within QTL intervals, the physical positions of SNP markers flanking the QTLs were searched in MSU Rice Genome Annotation (Osa1) Release 7. Genes contained in each QTLs were listed (Additional file 2: Table S3). To understand the roles of candidate genes in the mechanism of salinity tolerance, classification and annotation of candidate genes were inquired using the Panther Classification System (Mi et al. 2016).

## Additional files

Additional file 1: Table S1. Order and position of SNP markers in genetic map. (XLSX 234 kb) Additional file 2: Table S3. List of candidate genes contained in QTL intervals. (XLSX 84 kb) Additional file 3: Table S2. Comparison of 38 SNP calls by GBS and resequencing data. (XLSX 12 kb)

## Abbreviations

cM: centi-Morgan
GBS: Genotyping-by-sequencing
PVE: Phenotypic variance explained
QTL: Quantitative trait locus
RILs: Recombinant inbred lines
SDL: Segregation distortion loci
SIS: Salt injury score
SNP: Single nucleotide polymorphism
